# Supplementary material for: Are carbon emissions trading and green financial instruments synergistic? -Comprehensive quantitative research based on content analysis
Source: PLoS One. 2024 Mar 7;19(3):e0298601. doi: 10.1371/journal.pone.0298601 (PMC10919601; doi:10.1371/journal.pone.0298601)
Supplement: S1 Data — (ZIP) [file pone.0298601.s001.zip › Data/地级市Z_F工作报告.zip!/░▓╗╒/┘±╓▌╩╨/└┤╫╘╣1⁄2╓┌║┼í╛┬φ┐╦╩2╛▌═°í┐_2014.docx]

各位代表：
　　现在，我代表市人民政府，向大会报告工作，请予审议，并请市政协委员和其他列席人员提出意见。
　　一、凝心聚力，攻坚克难，经济社会发展稳中向好
　　刚刚过去的2013年，面对错综复杂的宏观经济形势，在中共亳州市委的坚强领导下，在市人大、市政协的监督支持下，市政府和全市人民一起，认真贯彻落实党的十八大和十八届三中全会精神，以科学发展为主题，以加快转变经济发展方式为主线，以稳中求进、好中求快、能快则快为主调，围绕市三届人大三次会议确定的目标任务，统筹推进稳增长、调结构、促改革、惠民生等各项工作，攻坚克难，开拓创新，经济社会呈现出良好发展态势，活力亳州、美丽亳州、幸福亳州建设取得新的成效。
　　初步预计，全市实现生产总值790亿元，同比增长10%；全部财政收入103.5亿元、增长21.6%，地方财政收入64.4亿元、增长35%；固定资产投资540亿元，增长26%；社会消费品零售总额345亿元，增长13.7%；城镇居民人均可支配收入22500元，增长10%；农民人均纯收入7530元，增长15%；节能减排主要目标任务全面完成。
　　过去的一年，受国内外经济下行压力的影响，我市个别经济指标增幅低于预期目标。但总体上看，一些主要经济指标增幅居于全省前列，经济增长处在合理较快的区间。1—11月份，8项指标增幅居全省前5位，其中，固定资产投资、房地产投资、限上消费品零售额、全部财政收入、地方财政收入5项指标增幅居全省前3位。尤为可喜的是：粮食生产实现“十连丰”，我市再次荣获“全国粮食生产先进市”称号，涡阳县、蒙城县荣获“全国粮食生产先进县”称号。一年来，我们主要做了以下工作：
　　（一）坚持稳中求进、创新驱动，经济发展后劲进一步增强。
　　力促实体经济稳步发展。及时出台促进经济持续健康较快发展的实施意见，认真落实国家结构性减税政策，加大企业帮扶力度，整合各类专项资金，大力支持民营经济和中小微企业发展。全年新增规模工业企业150家、总数达626家，实现规模工业增加值205亿元、增长15%；新增个体工商户34735家、私营企业3843家；减轻企业负担5.8亿元。
　　实施特色产业升级工程。成立十大产业发展战略研究推进工作领导小组，编制发展战略研究报告，开展十大产业专题培训，建立完善产业发展推进机制，特色重点产业不断壮大。现代中药产业呈集群化迅猛发展态势，产值同比增长21%；食品制造和农产品加工、汽车农机及零部件制造、劳动密集型装备制造等重点产业产值增速均在20%以上。
　　着力构建技术创新体系。实施省级以上各类科技计划项目53项，新获批认定高新技术企业5家、省级民营科技企业8家、创新型（试点）企业5家。申请专利1968件、其中发明专利764件，授权专利971件、其中发明专利105件；新增名牌产品5个、省著名商标25件，安徽古井贡酒股份有限公司荣获第二届省政府质量奖。建立院士工作站3个，古井集团设立全市首个国家级博士后科研工作站。成立浙江大学（亳州）技术转移中心，市科技创业服务中心入孵企业31家。谯城区荣获“全国科技进步考核先进县区”称号。
　　扎实推进园区快速发展。加快扩区升级步伐，增强园区承载力，5个省级开发区完成固定资产投资240亿元；新入园项目100个、开工63个，其中工业项目97个、开工60个；实现规模以上工业总产值271亿元。亳芜现代产业园起步区8平方公里的路网工程基本完成，新签约项目34个，协议总投资308亿元，开工亿元以上工业项目12个。
　　（二）坚持全面推进、重点突破，各项改革力度进一步加大。
　　加快行政管理创新。成立市城乡管理委员会，升格市城乡规划局，组建市招投标监管局，城乡规划建设和招投标监管工作进一步规范。合并原市经济开发区和市南部新区，管理体制进一步理顺。成立市现代中药产业发展管理委员会，下设办公室，与市药业发展局合署办公，服务药业经济发展的力量进一步加强。在市直行政事业单位组织开展“三清”工作，共清理资金18.4亿元、房产157.8万平方米、土地5368亩。
　　推进经济体制改革。完善市建投集团法人治理结构，推进市土地收储中心收储贷融资工作，组建市交投公司。市建投集团实现融资18亿元，拟发行的20亿元企业债券已上报国家发改委，药都地产实现融资10亿元，交投公司完成交通基础设施融资27亿元。成立亳州城镇发展股份有限公司，采取以市带县的新模式，推进新型城镇化项目建设和运营，有力支持了县域经济发展。
　　加强财税金融管理。稳步实施营业税改征增值税试点工作。调整理顺市对经济开发区、谯城区的财税征管体制。加快完善地方金融体系，蒙城、利辛湖商村镇银行、蒙城农商行、徽商银行利辛支行开业，涡阳村镇银行、利辛农商行已获国家银监会批准筹建，涡阳县农信社改制材料已上报国家银监会待批。典当、担保、金融租赁和小额贷款公司发展提速，银行、证券和保险机构开放力度进一步加大。全年新增贷款94.5亿元、增长22.1%，存贷比达56.2%、比年初提高3.7个百分点，其中新增存贷比82.5%，高于全省平均水平9个百分点。
　　深化重点领域改革。探索推进土地流转新机制，土地流转面积超过200万亩，涡阳县被列为全省农村综合改革试点县。基层医药卫生体制综合改革成果进一步巩固，县级公立医院改革全面推进。基础教育三项改革深入实施，中小学教师职称改革完成阶段性任务。
　　（三）坚持多措并举、扩大开放，投资拉动效应进一步发挥。
　　强力推进招商引资。坚持市级领导干部带头招商，组建驻长三角、珠三角、京津地区3个招商组，大力推进靶标招商、精准招商，实实在在地引进了一批大项目、大企业。全年实际利用市外内资819.6亿元、增长34.7%。投资5亿元的奇瑞重工农用机械装备制造、投资10亿元的太安堂人参加工和医药物流园、投资20亿元的海螺集团新型建材生产、投资20亿元的北汽集团现代农业装备生产、投资20亿元的江淮汽车零部件供应巢、投资23亿元的九州通现代中药物流产业园、投资60亿元的大连万达城市综合体、投资80亿元的九洲方圆药业、投资80亿元的科创集团健康产业城等一批重大项目纷纷落户亳州。
　　加快重大项目建设。坚持经济工作项目化、项目工作责任化，成立10个专业项目推进组，建立“一个项目、一名领导、一套班子”的工作推进机制。131个省“861”项目完成投资350亿元，336个市重点项目完成投资389亿元；全市在建亿元以上项目193个、完成投资308亿元，其中新开工亿元以上项目82个、完成投资135亿元。古井生态产业园、杉杉服饰、东方帝维等项目建成投产，广药集团大南药大健康双产业基地、修正药业、德通绿色食品等一批重大项目加快实施，利辛板集电厂主体工程今年4月有望开工。105国道涡河大桥、307省道亳涡段一级公路改造项目基本建成，济祁高速亳州段等项目开工建设，商杭高铁获国家发改委批复立项，皖江城际铁路网规划中的亳蚌铁路、徐淮铁路已通过国家发改委评审，亳州机场前期工作进展顺利。完成电网建设投资3.9亿元，220千伏赵桥变等4项输变电工程建成投运。一批打基础、管长远的重大项目加快推进，为我市经济社会快速发展奠定了坚实基础。
　　大力发展外向型经济。全市生产型外贸企业实现进出口额3.73亿美元，增长26.5%。新增外贸进出口实绩企业22家，进出口额超百万美元的企业达55家、新增5家，超500万美元的企业达29家、新增4家。全市实际利用外商直接投资4.7亿美元，增长31.4%。我市荣获“全国中药饮片出口城际第一名”称号，谯城区出口中药材质量安全示范区被认定为国家级出口食品农产品质量安全示范区。
　　（四）坚持培育主体、强基固本，现代农业基础进一步夯实。
　　大力培育新型经营主体。创新农业经营体制，着力培育种养大户，规范发展农民专业合作社，加快发展家庭农场。全市50亩以上种植大户发展到4172户，规模种植面积达146万亩；农民专业合作社发展到4489家，创建国家级示范社12家、省级示范社60家；家庭农场发展到305家。
　　突出发展优质高效农业。全年实现粮食总产447.4万吨，创建全省首个“吨粮市”活动取得阶段性成效，蒙城县实现“吨粮县”创建目标。新增设施蔬菜9万亩、蔬菜种植面积达190万亩，新增中药材种植3.2万亩、总面积达106万亩，新增无公害农产品产地11个、绿色食品原料基地6个，新增无公害农产品、绿色食品认证56个。新增各类规模养殖场、大户1774家，总数发展到2.02万家。规模养殖比重达75%，比去年提高2个百分点。新增国家级“一村一品”专业示范村（镇）1个、省级7个，省级休闲农业示范点1个。
　　着力加快农业产业化转型升级。新创建省级现代农业示范区2个，1个国家级、4个省级现代农业示范区核心区初步建成，市级以上农业产业化示范区实现产值220亿元；市级以上龙头企业发展到394家、新增64家，其中国家级4家、省级50家，实现年销售收入256亿元；全市规模以上农产品加工企业发展到325家、新增45家，完成产值450亿元。农作物耕种收综合机械化率达85%。
　　切实加强农村基础设施建设。全面完成年度农田水利基本建设任务，三县一区在全省第18届“江淮杯”竞赛中全部获奖；蒙城县芡河、利辛县西淝河及谯城区赵王河、油河、洺河、漳河治理工程基本完成，小农水重点县建设完成年度任务；建设高标准基本农田69.5万亩、水利受益区23.6万亩，补充耕地9000亩；全市农村饮水安全覆盖率达到64%。加固改造农村公路危桥129座，改造县乡公路200公里。
　　（五）坚持产城融合、建管并重，城乡面貌进一步改观。
　　高标准抓好规划编制。按照新型城镇化的要求，坚持“城乡全覆盖、规划一张图”，亳州全域规划编制工作扎实推进；按照决策民主化的方式，实行规划设计方案竞赛，进行多方案评审比选，建筑品位进一步提高；按照产城一体化的思路，坚持产业功能、城市功能、生态功能有机融合，宜居宜业的城镇发展格局初步形成。
　　大投入推进城乡建设。在市中心城区实施城建项目147个、完成投资95亿元，新建城市道路64.5公里，拆迁面积141万平方米，新开工建设还原小区120万平方米，10项重点工程完成年度任务，自来水普及率、污水集中处理率、燃气普及率分别达到80%、90%和87%。获批各类建设用地3.28万亩。涡阳南部新城、蒙城城南新区、利辛滨河新区建设步伐加快，修建道路约70公里。全力推进美好乡村建设，投入资金5.8亿元，开工项目375个，新建重点示范村26个。按照建设美丽小镇的要求，重点集镇规划建设开始启动，涡阳县高炉镇、蒙城县乐土镇、利辛县胡集镇和谯城区十八里镇等乡镇较好发挥了示范引领作用。
　　严管理解决突出问题。出台进一步加强城市管理工作的意见和配套措施，大力推进城市网格化、责任化、信息化、精细化管理。强力推进拆违控违，拆除违章建筑31.3万平方米；依法取缔非法客运三轮车5646辆，新增公交车150辆，优化公交线路14条；全面推进农村清洁工程，清理垃圾16.5万吨，清理河道3587公里，拆除违章搭建物2.4万处。人民群众反映强烈的私搭乱建、交通拥堵和环境脏、乱、差现象初步得到遏制。
　　（六）坚持优化结构、提质增效，第三产业进一步繁荣。
　　大力发展商贸物流业。全力推进商贸重点项目建设，投资15亿元的亳州康美国际中药城一期工程投入使用，投资22亿元的大唐国际城、投资12亿元的昌升药包材市场等项目进展顺利，涡阳万隆城市综合体、蒙城广运现代物流服务中心、利辛金龙商贸港、谯城天泉医药物流等项目加快建设。深入实施“万村千乡市场工程”和“新网工程”，积极推进“五位一体”便民服务体系建设，新建、改造农家店61个、配送中心和乡镇商贸中心5个、农贸市场10个，新建便利店45个、早餐网点50个，新培育家政服务龙头企业4个。全市净增限额以上流通企业99家，总数达446家。
　　积极发展文化旅游业。加大旅游景点、景区建设力度，曹操地下运兵道出入口改造、南京巷钱庄改扩建、华仑国际文化广场一期工程基本完工，花戏楼景区、北关历史街区改造工程和道德中宫、薛阁塔、八角台遗址修缮保护工程加快推进，蒙城竹海湾生态旅游度假村项目顺利实施，总投资50亿元的木兰魔幻大世界即将开工建设。提升旅游休闲、宾馆服务产业功能，创建3A级以上旅游景区3家、3星级以上农家乐7家、3星级以上旅游饭店4家。全市旅游总收入78亿元，增长20.4%。
　　培育壮大新兴服务业。发展会展经济，成功举办2013年国际（亳州）中医药博览会暨第29届全国（亳州）中药材交易会。积极发展电子商务，“药通网”被批准为全国首批中药材电子商务示范平台。培育规范房地产市场，全市完成房地产开发投资154亿元、增长60.5%，商品房销售面积265.7万平方米、增长44%。养老、托幼、文化、健身、物业管理等便民服务业加快发展，会计、审计、法律、工程、评估、咨询、劳动力等中介服务业规范化水平进一步提升。
　　（七）坚持以人为本、民生为先，社会建设进一步加强。
　　民生保障水平稳步提升。按照“保民生、保发展、保运转”的思路，继续加大对民生工程和社会事业的投入。全市完成财政支出205.4亿元、增长15.9%，其中，用于改善民生方面的支出166.4亿元，占全年财政支出的81%。投入资金80.2亿元，全面完成31项民生工程。大力推进重点人群就业和创业带动就业，新增城镇就业6.14万人、转移农村富余劳动力10.1万人。实现城乡居民社会养老保险制度全覆盖，五项社会保障完成年度任务。企业退休人员养老金人均月增加153元，城市居民、农村居民最低生活保障标准分别提高10.2%和11.3%，农村五保户供养补助、年度残疾人生活救助标准均提高10%，政策范围内新农合、城镇职工医保住院费用报销比例进一步提高。新建和改扩建敬老院27所，新增床位2932张，农村五保老人集中供养能力提高到59%。建设各类保障性住房和棚户区改造住房3.8万套、295万平方米。
　　公共服务能力不断提高。全市财政性教育经费投入42亿元，占地区生产总值的5.3%。学前教育三年行动计划全面完成，毛入园率达76.5%；义务教育阶段标准化学校建设超额完成省下达任务，千所村小提升工程加快推进；普通高考本科达线率53.6%，超过全省平均水平9个百分点。亳州幼师新校区一期工程建设加快，亳州中药科技学校二期工程启动实施，亳州师专升格本科工作取得新的进展。市人民医院顺利通过“三甲”复审，县级公立医院全部达到“二甲”水平，80%以上的乡镇卫生院和社区卫生服务中心设置中医科，市精神病医院、市全科医师培训中心主体工程完工。市社会福利中心和3所县级流浪乞讨人员救助站投入使用。市图书馆、蒙城博物馆主体工程基本建成，建安文学馆正在进行设计施工招标。广泛开展全民健身运动，华佗五禽戏普及面进一步扩大，涡阳县被国家体育总局评为“全国群众体育先进单位”。人口自然增长率8.47‰，低生育水平继续稳定。老龄、慈善、红十字会和残疾人事业取得新进步。物价、统计、气象、地震、科协、档案、地方志、广播电视、新闻出版、妇女儿童、民族宗教、外事侨务、对台事务、扶贫开发、无线电管理、机关事务管理等工作均取得显著成绩。
　　生态文明建设持续加强。实施千万亩森林增长工程，完成植树造林12.03万亩，建设绿色长廊600公里、长防林2.2万亩，新建完善农田林网85万亩，创建森林城镇10个、森林村庄35个。建设城市绿道51公里，创建国家级休闲农业示范点1个、省级2个，创建省级休闲农业示范县1个、省级生态镇2个、省级生态村4个。蒙城县被全国绿化委评为“全国绿化模范县”。
　　社会大局保持和谐稳定。完善信访工作机制，建成群众来访接待中心，妥善化解了一批信访积案，信访秩序进一步好转。修订完善应急预案，健全应急管理体系，加强消防安全工作，突发事件应急处置能力不断提高。深入推进平安亳州建设，全面完成“六五”普法中期任务，依法预防和打击各类违法犯罪活动，群众法律意识和安全感不断增强。落实安全生产责任，安全生产形势基本稳定。强化食品药品安全监管，药品药材“两打两建”工作取得阶段性成效。不断加强国防教育、国防动员、双拥优抚和人民防空工作，推进国防后备力量建设，市及三县均荣获省级“双拥模范城”称号。
　　各位代表，在促进经济社会发展的同时，我们始终紧紧围绕为民务实清廉的要求，高度重视并切实加强政府自身建设。严格执行中央八项规定和省委、市委三十条具体措施，坚决反对形式主义、官僚主义、享乐主义和奢靡之风，在作风建设方面取得积极成效。自觉接受人大法律监督、工作监督和政协民主监督，依法执行人大决议决定，全年办复人大代表议案8件、建议286件，政协委员提案370件，满意率达95%以上。大力弘扬少说多做、真抓实干的作风，把察实情、出实招、求实效作为行为准则，力行开短会、发短文、办实事，切实精简文件、会议和检查评比活动，建立政府重点工作每月一督查、每月一通报机制，行政效能进一步提高。积极整合为民服务平台，加强12345政务服务直通车和市政府门户网站建设，推进政府信息公开，工作透明度进一步提升。切实加强廉政建设，继续实施“四项安全”工程，严格落实廉洁从政各项规定，强化行政监察和审计监督，坚决查处腐败案件，政府公信力进一步增强。
　　回顾不平凡的2013年，面对严峻挑战和竞争压力，全市人民同舟共济、风雨兼程，携手走过了一段难忘的岁月，奋斗的足迹历历在目。我们不会忘记，每一项工作的扎实推进，每一个难题的成功破解，都离不开人民群众的理解和支持，都凝聚着广大干部的智慧和力量。在此，我谨代表市人民政府，向全市广大工人、农民、知识分子，向全体干部和离退休老同志，向人大代表、政协委员，向各民主党派、工商联、各人民团体，向驻亳部队、武警官兵和政法干警，向中央、省驻亳各单位，向所有来亳投资者和建设者，以及关心支持亳州发展的各界人士，致以崇高的敬意，表示衷心的感谢！
　　我们也清醒地看到，全市经济社会发展中还存在不少困难和问题。主要是：历史形成的产业结构不够合理、经济总量和人均水平在全省落后的局面还没有从根本上改变；经济下行压力加大，地区生产总值、城镇居民人均可支配收入和进出口总额增幅没有达到预期目标；一些企业经营困难，工业增速明显回落，农业基础还不牢固，现代服务业刚刚起步；城镇化发展质量不高，城市管理还存在一些薄弱环节，违法违章建设依然存在，脏、乱、差现象还没有得到彻底根治；形式主义、官僚主义、享乐主义和奢靡之风在一些地方还不同程度地存在，少数干部作风不实、效率不高、为政不廉。对此，我们将采取针对性措施，切实加以解决。
　　二、稳中求进，改革创新，扎实做好2014年各项工作
　　2014年是深入贯彻落实党的十八届三中全会精神、全面深化改革的重要一年，是推进经济持续健康较快发展、完成“十二五”规划目标任务的关键一年。做好今年的政府工作，意义十分重大。
　　当前，世界经济缓慢复苏，增长格局深度调整；全国经济稳中有进、稳中向好，正进入增长速度换挡期、结构调整阵痛期、前期刺激政策消化期；全省经济增势强劲，持续向好的基本面更加巩固，正处于大有作为的快速发展期；我市工业化、城镇化和农业现代化加速推进，综合实力和内生动力进一步增强，后发优势开始显现，发展前景十分广阔。党的十八届三中全会开启了新一轮改革开放的历史征程，必将在亳州大地释放出巨大的发展红利。面对新的形势，我们要准确把握中央和省委、市委对经济形势的新判断，紧密结合实际，增强机遇意识、责任意识和进取意识，奋力开创亳州经济社会发展新局面！
　　今年政府工作的总体要求是：坚持以科学发展观为指导，全面贯彻党的十八大、十八届三中全会和中央、全省经济工作、城镇化工作会议精神，以稳中求进、好中求快为基调，以改革创新、扩大开放为动力，激发市场活力，加快四化进程，统筹城乡发展，倾力改善民生，促进经济持续健康较快发展，保持社会大局和谐稳定，在建设活力亳州、美丽亳州、幸福亳州上迈出更加坚实的步伐！
　　经济社会发展的主要目标是：地区生产总值增长10%左右，财政收入增长16%以上，固定资产投资增长20%以上，社会消费品零售总额增长12%以上，进出口总额增长10%以上，城镇居民人均可支配收入增长10%左右，农民人均纯收入增长14%以上，城镇登记失业率控制在4.2%以内，节能减排完成省政府下达的年度目标任务。
　　围绕上述目标，重点做好十个方面的工作：
　　（一）以全面深化改革为统领，构筑体制机制新优势。
　　紧紧围绕使市场在资源配置中起决定性作用，着力在重要领域和关键环节改革上取得实质进展。
　　增强市场主体活力。深化国有企业改革，把发展混合所有制经济作为国企改革方向，有序推进国有企业重组合并、做大做强。完善古井集团、市建投集团法人治理结构，建立符合企业功能定位的考核评价指标体系和规范合理的薪酬制度。强化国有资产管理，巩固市直行政事业单位“三清”成果，在三县一区全面推开。大力发展非公有制经济，全年新增个体工商户22000户，新增私营企业2400家。
　　深化财税金融改革。改进预算管理制度，年内实现市县级部门预决算公开。把政府性债务纳入全口径预算管理，编制权责发生制的政府综合财务报告，做好债务风险防控和化解工作。理顺市区财政收支征管体制，严禁收入混库。继续做好“营改增”扩围试点，清理规范市县税收优惠政策。加快金融改革创新，确保利辛、涡阳农商行和涡阳、谯城村镇银行上半年挂牌。积极发展保险、证券、融资租赁、典当公司等金融和类金融机构，规范发展市县融资平台和小额贷款公司、融资担保公司，谋划设立新型城镇化建设基金。加快企业上市培育，积极推动符合条件的企业进入“新三板”和安徽省中小企业股权交易中心挂牌融资。
　　统筹推进城乡改革。加快推进户籍制度改革，有序推进农业转移人口市民化，稳步实现城镇基本公共服务常住人口全覆盖。积极争取列入国家、省“工矿废弃地复垦”、“人地挂钩”、“农村宅基地”和国家新型城镇化改革试点。加快推进土地承包经营权确权登记颁证工作，引导土地向新型农业经营主体流转。创新农村公共基础设施建设和管理机制，积极推进小型农田水利管理改革创新试点工作。
　　加快政府职能转变。进一步简政放权，承接落实好中央和省下放的审批事项，积极推进市级行政审批项目清理，切实做好工商登记“先照后证”工作。加快推进机构改革和部门职责整合，1月底前完成工商、质监行政管理体制调整和食品药品监管体制改革，年底前完成卫生与人口计生机构整合。规范公共资源交易管理，加大对招投标和政府采购的监管力度。
　　抓好社会事业改革。深入推进教育领域综合改革，加快完善中小学教师招聘、职称评定、资格考试和定期注册制度。创新公共文化运行机制，推动政府部门由办文化向管文化转变。深化医药卫生体制改革，健全基层医疗卫生机构补偿机制和运行机制，探索推行药品阳光集中配送新模式。按照国家、省统一部署，及时落实完善生育政策。
　　（二）以加快转型升级为重点，拓展开发开放新空间。
　　按照经济社会发展向资源节约型、环境友好型转变的新要求，着力抓好项目建设、招商引资、园区建设和对外开放工作。
　　实施项目建设转型升级工程。以项目建设为抓手，坚持扩大总量与优化结构并重，围绕重点产业、重要基础设施、重大民生项目三个重点，落实领导包保、分类调度和月度通报制度，努力扩大有效投入。全年新开工超亿元项目100个以上，建成超亿元项目50个以上；新谋划储备亿元以上项目400个，总投资1000亿元以上。加快济祁高速亳州段、307省道涡阳至蒙城段一级公路、202省道改造建设，推进商杭高铁、亳州机场、引淮济亳、涡河航道疏浚、涡北煤矸石综合利用电厂等项目前期工作，积极谋划争取贯穿皖北、连接中原经济区的城际铁路项目。
　　实施招商引资转型升级工程。把招商引资提质提效作为经济发展的第一要事，更加注重招大引强，更加注重招才引智，坚决摒弃拼政策、拼资源、拼土地的做法，坚决防止引进落后产能和污染项目。密切关注高新技术、新兴产业前沿动态，充分利用小分队招商、驻点招商、产业招商、借会招商等有效方式，着力拓展与央企、省企、知名民企合作空间，力争在重点发展产业、重大社会事业、重要基础设施等领域引进一批大项目、好项目。严格执行“六库合一”制度，完善招商引资考核办法，建立健全激励机制，提高项目成功率。全年引进超亿元项目100个以上，其中工业项目60个以上；利用市外内资同比增长20%以上。
　　实施园区建设转型升级工程。加快机制和管理创新，进一步挖掘潜力、激发活力，优化功能布局，推动产城一体，提升产业承接规模、质量和集中度。加大5个省级开发区内涵提升力度，加快亳芜现代产业园扩区步伐，积极推进省级开发区升格为国家级开发区。加快现代中药、劳动密集型装备制造、电子信息、农产品加工等专业园区建设，推动产业集聚。继续抓好标准化厂房建设，探索企业进退机制，促进土地集约节约利用，提高园区投入产出效益。
　　实施外向型经济转型升级工程。坚持以开放倒逼改革，统筹做好外贸、外经、外资等各项工作。实施市场多元化战略，支持企业开拓国际市场，扩大对外贸易，进出口总额实现4.7亿美元。抓好进出口企业孵化工作，新增进出口实绩企业20家。积极争取外国政府和国际组织贷款，全年实际利用外商直接投资增长15%以上。全力支持企业“走出去”发展，力求在更高层次、更宽领域参与国际分工与合作。
　　（三）以推进新型工业化为核心，促进发展方式新转变。
　　突出工业强市战略，启动实施“1615培育提升计划”，聚焦重点产业，延伸产业链条，打造产业集群，以此牵引经济整体发展。
　　聚焦发展重点产业。紧紧围绕现代中药千亿元发展目标，加快建设亳州现代中药产业园区，全力推进科创健康产业城、九洲方圆药业、太安堂药业、修正药业、广药集团项目建设，确保年内实现医药制造业产值170亿元。改造提升白酒、食品制造及农产品加工、煤电煤化工等传统产业，加快推进古井配套产业园、五得利面粉等项目建设，力争板集2×100万千瓦坑口电厂形成实物投资量。加快培育新兴产业，强力推动汽车农机及零部件、电子信息、劳动密集型装备制造业集聚发展，做大做强江淮安驰、联滔电子、奇瑞重工等龙头骨干企业，加快建设北汽集团现代农业装备生产、江淮汽车零部件供应巢项目。重点支持100户有发展潜力的小微企业，培育发展100户规模以下的成长型企业，加快建设100个重大工业项目，确保全年新增规模工业企业100家，规模工业增加值增长15%以上。
　　提高自主创新能力。坚持创新驱动，着力培育一批高新技术企业和创新型企业，新增高新技术企业5家、省级创新型（试点）企业4家，高新技术产业产值和增加值均增长20%以上。完成发明专利申请700件；新增名牌产品4个、省著名商标18件。古井集团和浩翔农牧院士工作站力争年内挂牌成立。
　　加大实体经济政策支持力度。深入开展“千名干部联系服务千家企业”活动，落实和完善稳定经济增长政策，切实减轻企业税费负担，突出解决好小微企业融资难、用地难、用工难等问题。建立扩大信贷投放激励机制，鼓励引导金融机构加大对“三农”、小微企业的支持，确保全年新增贷款高于上年。
　　（四）以促进农民工市民化为关键，探索城镇化发展新路径。
　　坚定不移地推进以人为核心的新型城镇化建设，提升城镇发展质量，力争城镇化率提高2个百分点。
　　优化城镇发展布局。贯彻落实《国家新型城镇化规划》，按照城市在田园之上、田园在城市之中的理念，突出清水、绿树和文化三大特色，着力打造“田园城市”。制定完善我市新型城镇化发展规划，突出解决好“人往哪里去、钱从哪里来、土地怎么用、城镇怎么管”四大问题，重点解决已进城常住农业转移人口落户问题。根据资源环境承载能力，突出组团式发展，促进中心城区与三县县城共同发展，三县县城与中心集镇融合发展，美丽小镇与美好乡村协调发展。以建设美丽小镇为重点，每个县区每年选择2—3个乡镇政府所在地，按照微小城市标准规划建设。坚持严守底线、严控增量、盘活存量，促进土地节约集约利用，合理确定城镇开发强度，提高城镇建设用地利用率。
　　提高城镇建设水平。城市建设水平是城市生命力所在，我们将按照中央城镇化工作会议部署，在推进新型城镇化中体现尊重自然、顺应自然、天人合一的理念，让居民“望得见山、看得见水、记得住乡愁”。
　　今年，在市中心城区安排建设项目247个、总投资87.4亿元，其中续建项目183个、投资56.6亿元，新建项目64个、投资30.8亿元。突出抓好10项重点工程：1.加快保障性住房和安置还原房建设。2.实施城区水系治理和公园建设工程。3.实施新区路网完善和高速公路出入口改造工程。4.实施老城区基础设施完善工程。5.开工建设涡河人民大桥工程。6.开工建设市文化馆、职工之家等文化工程。7.开工建设市城市规划展览馆、城市建设档案馆和体育馆，启动市博物馆新馆建设前期工作。8.实施市人民医院二期、市养老服务中心和流浪乞讨人员安置中心建设工程。9.建设国家中药材质量监督检验中心和市农产品质量安全检测中心。10.实施城市生活垃圾处理工程。同时，加快数字城管和城区智能交通体系建设，大力发展城市公共交通，推进市客运西站和公交港湾建设，优化公交运行线路，新增一批公交车、出租车，加强停车场位建设，满足群众出行需求。
　　推进城市管理机制创新。健全城市管理组织体系，落实网格化责任机制，建立常态化投入机制，严格制度化奖惩机制，推进管护作业市场化，不断提高城市管理水平。抓好市容市貌综合治理，落实城市环境卫生责任制，规范户外广告和建筑垃圾、渣土运输管理，坚决查处各类违法违章建设。进一步抓好城区交通秩序专项整治，巩固治理非法客运三轮车工作成果，坚决打击电动四轮车和“黑出租”非法营运行为。
　　（五）以稳定粮食生产为前提，实现“三农”工作新跃升。
　　深入贯彻落实中央农村工作会议精神，按照“稳定政策、改革创新、持续发展”的总要求，全面深化农村改革，加快推进农业现代化。
　　提高农业综合生产能力。始终把稳定粮食生产、保障农产品供给作为农业发展的首要任务，继续抓好“吨粮市”建设，大力推进农业结构调整，深入开展粮食整建制高产创建和蔬菜标准园创建活动，提升中药材标准化种植水平，粮食总产稳定在450万吨以上、蔬菜面积达到195万亩。大力发展畜禽标准化养殖，力争规模养殖比重达到78%。加快构建农产品全程质量控制系统和追溯制度，新增无公害农产品产地、绿色食品原料基地12个，新增“三品一标”农产品24个。支持农业保险提标扩面，为农业生产提供保障。
　　培育新型农业经营主体。推进多种形式的规模经营，力争年内家庭农场发展到1000家，新增695家；农民专业合作社发展到4800家，新增各级示范社160家。加大对农业龙头企业的扶持力度，着力培育100家规模大、竞争力强的龙头企业，力争亿元以上企业超过80家。加快推进现代农业示范区建设，力争市级以上产业化示范区产值超过260亿元。支持农机大户、农机专业合作社开展订单作业、承包作业和一条龙作业，农作物耕种收综合机械化率达86%以上。
　　强化农业基础设施建设。进一步改善以水利为重点的农业基础设施，全面完成西淝河、漳河、洺河等中小河流治理工程及7座中型水闸除险加固工程；大力实施“5588”行动计划，更新改造小型泵站25座、总装机容量1380千瓦，新建和加固小型水闸48座，改造中型灌区2处，扩挖塘坝2192处，整治中小河沟607条，修复和新建机井8587眼；新增和改善有效灌溉面积32万亩，建设高标准农田水利受益区8万亩。加固改造农村公路危桥140座，改建农村公路200公里，建设气象防灾减灾标准化乡镇和示范村8个。
　　全面推进农村清洁工程。围绕“凡土必绿、凡地必净、凡水必清、凡村必洁”的目标，在全市14736个村庄、89个集镇全面实施农村清洁工程。全年清理垃圾20万吨以上，清理河道4000公里以上，垃圾收集点达到5万个。
　　切实抓好美好乡村建设。建立农村规划管理新机制，提升农村建设水平，逐步改变千村一面、千户一貌的状况。推进28个重点示范村建设，完善中心村道路、电力、供排水等基础设施，实施各类建设项目420个，总投资6.3亿元。在示范村建设中，要慎砍树、不填塘、少拆房，切实做到宜建则建、宜改则改。
　　（六）以扩大消费需求为带动，实现第三产业新突破。
　　充分发挥消费对经济社会发展的拉动作用，认真落实刺激消费增长的政策措施，带动第三产业快速发展，年内新增限额以上商贸企业70家。
　　着力提升服务业发展水平。加快推进万达城市广场、大唐国际城等大型商业综合体建设，构建标志性城市商业集聚圈。扶持引导九州通、九洲方圆等专业物流园区建设，打造区域性中药材物流中心。支持发展邮政快递业，构建以快件分拨中心为核心的快递园区。着力促进电子商务等新型交易业态发展，年内争取建成亳州中药材商品交易所。积极发展会展经济，办好2014年国际（亳州）中医药博览会暨第30届全国（亳州）中药材交易会。发展总部经济，规范发展法律、会计、审计、咨询等中介服务业。
　　努力开拓城乡消费市场。大力实施“万村千乡市场工程”和“新网工程”，深入开展“农超对接”、“农商对接”活动，新建和改造农家店120家、农村社区综合服务中心（社）20家。启动市区标准化菜市场建设和改造工程，重点推进板桥口、涡北还原小区等菜市场建设。加快发展健康养老产业，促进养老服务与医疗、家政、健身等相关领域互动发展。积极推动信息网络消费，加快建设城市社区、农产品电子商务平台。稳步发展房地产业，继续抓好保障性住房和普通商品房建设。整顿和规范市场秩序，依法严厉打击制售假冒伪劣商品、欺行霸市等违法行为，进一步净化消费环境。
　　加速发展现代旅游业。围绕建设新皖北旅游中心的目标，发挥国家历史文化名城、中国优秀旅游城市优势，推动历史文化游与现代科技游相结合、休闲娱乐游与养生保健游相结合，促进文化旅游业与中药产业、白酒产业、现代农业融合发展。今年，重点加快木兰魔幻大世界、中华药都养生园、蒙城竹海湾生态旅游度假村等项目建设。大力培育A级景区、星级农家乐，全年力争创建3A级以上景区2家、3星级以上农家乐5家、3星级以上旅游饭店3家。
　　（七）以创建文明城市为抓手，推进文化建设新发展。
　　广泛开展丰富多彩的文明创建活动，充分激发文化创造活力，全面推进文化事业、文化产业繁荣发展。
　　加强思想道德建设。倡导践行“富强、民主、文明、和谐，自由、平等、公正、法治，爱国、敬业、诚信、友善”的社会主义核心价值观，集聚实现中国梦的强大正能量。深入实施公民道德建设工程，切实加强市民素质教育，扎实做好“中国好人”、“安徽好人”、“亳州好人”评选工作。广泛开展精神文明创建活动，抓好省级文明城市、文明村镇、文明单位、文明家庭创建工作。大力宣传亳州精神，凝聚全市人民热爱亳州、发展亳州的精神力量。加强对社会热点的舆论引导，营造昂扬向上、和谐奋进的社会氛围。
　　提升公共文化服务水平。加快市县图书馆、文化馆、博物馆等公益性文化设施建设，做好公共文化场馆免费开放工作，推动公共文化服务体系向基层延伸。深入实施文化惠民工程，广泛开展群众性文体活动和全民阅读活动。加强文化遗产保护、传承和利用，推进第一次可移动文物普查工作。鼓励社会力量、社会资本参与公共文化服务体系建设，让人民群众广泛享有基本公共文化服务。
　　繁荣文化市场。充分发挥老庄文化、中医药文化、酒文化等资源优势，促进文化与科技融合，大力培育文化市场主体，发展新兴文化业态，加快建设一批特色文化产业项目。实施文艺精品工程，扩大对外宣传和交流，不断提升亳文化的知名度和影响力。
　　（八）以实现生态文明为目标，建设美丽幸福新家园。
　　坚持绿色低碳可持续发展，正确处理发展经济与保护生态环境的关系，让亳州的天更蓝、地更绿、水更清、环境更优美。
　　推进资源节约集约利用。倡导全民节能，严格项目节能评估和审查。加大企业技改力度，组织实施重点企业节能改造工程。落实最严格的耕地保护制度和水资源管理制度，大力推进循环经济和资源综合利用，严格地下水管理和保护，支持节能低碳产业和新能源、可再生能源发展。
　　切实抓好环境保护工作。认真落实省大气污染防治行动计划实施方案，确保今年我市PM10年均浓度比2012年下降2.1%以上。完善环境监测体系，完成市区空气自动监测站升级改造工程，每个县至少建成1个空气自动监测站。全面治理市区工业污染、机动车尾气、建筑工地等重点污染源，今明两年全面取缔城区燃煤锅炉，确保主要污染物持续均衡削减。凡主要污染物或重金属长期不能稳定达标排放的企业，坚决予以关闭。加强固体废弃物的处置管理，做好秸秆转化利用工作。加快污水处理厂建设，确保市南部新区污水处理厂投入使用。继续推进农业源污染减排工程建设，完成60家规模化养殖场（小区）污染治理任务。
　　全面提升城乡绿化水平。继续加快实施“千万亩森林增长工程”，在市中心城区启动创建国家级园林城市，推进三县创建森林县城，抓好森林乡镇、森林村庄创建工作。切实抓好年度植树造林，以增加冬季绿色、力争四季有彩为目标，完成人工造林面积12.5万亩，新建和完善农田林网85万亩，建设绿色长廊590公里，力争森林覆盖率达到18.5%。
　　扎实开展“三线三边”环境整治工作。将“三线三边”环境整治作为文　　明创建的重要内容，列入年度工作重点，纳入目标管理考核，建立一月一督查、一月一通报制度。市财政设立奖励资金，实行以奖代补，做到奖优罚劣。
　　（九）以保障和改善民生为目的，让人民共享改革发展新成果。
　　致力保障和改善民生，积极推进社会建设，让全市人民共享改革发展新成果、共创和谐幸福新生活。
　　推动实现更高质量的就业。最大的民生就是就业。把保就业作为稳增长的底线，一手抓援企稳岗，一手抓创业带就业，多渠道创造就业岗位。重点做好高校毕业生、城镇困难人员、退役军人等重点群体就业工作。加大就业和创业培训力度，推进创业孵化载体建设，新增城镇就业岗位3万个，转移农村富余劳动力7.6万人。
　　提升民生保障水平。扎实做好民生工程各项工作，确保完成省下达的目标任务。在上年的基础上，今年将城市、农村低保标准分别再提高10%和15%以上，农村五保供养标准提高10%以上。加大敬老院建设力度，新增床位1500张，五保集中供养能力达65%以上。加快健全覆盖城乡居民的社会保障体系，逐步提高社会保险、社会救助、社会福利水平。推进廉租房、公租房并轨运行，完善建设、分配、管理、退出机制，完成全年保障性住房建设任务。加强价格监督机制建设，保持物价水平基本稳定。
　　统筹发展各项社会事业。制定实施学前教育三年提升计划，在中小学开展国学经典教育。继续推进义务教育阶段学校标准化建设，做好中小学校长、教师轮岗工作。加快市职教园区建设，确保亳州幼师新校区建成使用。加大对亳州师专升格本科的投入力度，力争取得实质性进展。支持亳州职业技术学院与安徽中医药大学开展交流合作，确保本科班实现招生、中药执业药师培训班开班。设立困难家庭子女就学扶助专项资金，努力不让一个困难家庭孩子失学辍学。巩固基本医保覆盖面，提高基本医疗保障和服务水平，继续实施中医药服务能力提升工程。稳定低生育水平，强化流动人口服务创新，人口自然增长率控制在9‰以内。加快建设现代服务型统计，扎实做好第三次经济普查工作。完善覆盖城乡的全民健身公共服务体系，大力推广普及华佗五禽戏，积极参加省第十三届运动会。加大扶贫开发力度，做好民族、宗教工作，保障妇女儿童合法权益，支持残疾人事业发展。加强外事、侨务、对台、防震减灾、无线电管理和机关事务管理工作，推动档案、地方志、红十字会、老龄事业稳步发展。
　　（十）以维护群众利益为宗旨，巩固社会和谐新局面。
　　着眼于维护群众根本利益，不断提高社会治理水平，确保人民安居乐业、社会和谐稳定。
　　创新社会治理。改进社会治理方式，完善人民调解、行政调解、司法调解联动工作体系，加强社区矫正和法律援助工作，建立调处化解矛盾纠纷综合机制。改革社会组织登记管理体制，激发社会组织活力。加快推进城乡社区建设，提升社区服务功能，开展第九届村委会换届选举工作。充分发挥工会、共青团、妇联等人民团体在社会治理中的重要作用。
　　做好信访工作。坚持以群众工作统揽信访工作，认真落实信访工作各项制度，深入开展党政领导干部公开接访、带案下访、重点约访。进一步加强基层信访工作，积极开展网上信访，畅通诉求表达渠道，依法规范信访秩序。严格实行“三位一体”包案化解和工作倒逼机制，解决群众合理诉求，集中化解一批信访积案。
　　维护社会稳定。按照建设法治中国的要求，加快法治亳州建设，深入实施“六五”普法规划。继续推进平安亳州建设，完善社会治安防控体系，进行社会稳定风险评估，加强国家安全工作，依法预防和打击各类违法犯罪活动。强化食品药品安全监管，严厉打击食品药品违法生产经营行为，巩固药品药材“两打两建”成果，加强中药材市场监管，保障人民群众饮食用药安全。健全安全生产责任体系，抓好隐患排查治理和重点行业、重要领域集中整治工作，严防重特大事故发生。强化应急平台和专业队伍建设，做好消防安全工作，提高应急处置能力。扎实推进国防动员、国防教育、人民防空、优抚安置和双拥共建工作，巩固发展军政军民团结的大好局面。
　　三、为民施政，勇于担当，全面加强政府自身建设
　　经济社会发展的新形势，全面深化改革的新任务，要求我们加快推进治理体系和治理能力现代化。我们将以为民务实清廉为总要求，加快转变职能，创新治理方式，不断增强政府公信力和执行力，努力建设法治政府和服务型政府。
　　（一）履职尽责，提高行政效率。正确处理政府与市场、政府与社会的关系，深入推进政企、政资、政事、政社分开，把政府工作重点转移到创造良好发展环境、提供优质公共服务、维护社会公平正义上来。着力提升政府治理能力，坚持有所为有所不为，推行“负面清单”管理模式，创新公共服务供给方式，满足群众多元化服务需求。强化政府公共服务、市场监管、社会治理、环境保护等职责，更加注重劳动就业、居民收入、社会保障、人民健康状况，更好发挥政府为民服务作用。严格政府绩效管理，持续推进机关效能建设，深入开展“发展环境优化年”和民主评议百名科长活动，着力整治慵、懒、散、拖、贪现象。
　　（二）依法行政，规范政府行为。严格按照法定权限、法定程序履行职责、行使职权。坚持依法科学民主决策，建立健全重大决策的规则和程序，完善落实调查研究、公众参与、专家论证、合法性、廉洁性审查和集体决策制度，不断提高决策透明度和公众满意度。自觉接受人大法律监督、工作监督和政协民主监督，建立健全与人大、政协定期沟通协商制度，重大决策出台前向本级人大报告并听取政协意见。广泛听取各民主党派、工商联、无党派人士和各人民团体意见，高度重视司法监督、舆论监督和社会监督。完善政务公开制度，加强政府信息公开平台建设，有序推进决策公开、管理公开、服务公开、结果公开，及时主动公开涉及公众利益的信息，向群众说真话、交实底，让群众看得懂、用得上。
　　（三）改进作风，树立良好形象。扎实开展群众路线教育实践活动，严格执行中央八项规定和省委、市委三十条规定。围绕反对形式主义、官僚主义、享乐主义和奢靡之风，加快体制机制建设。全面贯彻《党政机关厉行节约反对浪费条例》，严格执行会议公文办理、公务接待、出国出境、办公用房、公务用车等管理规定。认真落实党风廉政建设责任制，深入推进廉政风险防控管理，依法加强对公共资金、国有资产、国有资源的行政监察和审计监督，确保生产安全、工程安全、资金安全和干部安全。继续开展“八千干部联系帮扶万名困难群众”活动，建立健全深入了解民情、充分反映民意、广泛汇集民智的工作机制。
　　（四）锐意进取，推动工作落实。始终保持奋发有为的精神状态，把握新形势、新判断，强化目标导向，勇于担当，敢于负责，确保各项任务落到实处。始终保持攻坚克难的进取意识，正视新问题、新矛盾，强化问题导向，敢于啃硬骨头、敢于涉险滩，努力破解发展中的一个个难题。始终保持雷厉风行的工作作风，面对新任务、新要求，强化责任导向，一招不虚抓发展，踏石留印抓落实，以实实在在的业绩赢得群众的信任和支持。
　　各位代表！改革开放的新征程已经开启，加速崛起的新任务催人奋进。让我们在中共亳州市委的坚强领导下，紧紧依靠全市人民，励精图治，锐意进取，为加快建设活力亳州、美丽亳州、幸福亳州，全面建成小康社会而努力奋斗！
